# Indents

**Published:** 1916-05

**Authors:** 


### INDENTS

**☞ Brief Articles of Technical or Scientific Value will receive notice and comment. Contributions invited.**

*Comprehensive Dentistry.* The prestige and income of many a dentist suffers because he is not comprehensive enough in his daily work.

It is altogether too true that the average dentist just "putters" and "patches" when it comes to his everyday routine work.

A patient comes to the office and he sees one or two teeth to be filled or possibly a bridge to be inserted, and he does that small amount of work, charges a small fee for it and sends the patient away as quickly as possible so that he can get to work on the next patient who perhaps is waiting in the reception room.

This average dentist that we have in mind does not take the time to see the possibilities of the first case. Consequently, he is not fair to himself nor does he render to the patient the full professional service which it is his privilege and his duty to provide and which would result in a larger financial reward. He fails to be comprehensive in his work. He does not see the mouth as a whole, or, as someone has said. "He can't see the forest for the trees."

To most people, a filling is a filling, and a gold crown is a gold crown. They do not recognize any difference. The intelligent, wide-awake, progressive dentist should make it his business to make his patient understand the difference in so far as he is able.

When a new patient is received, there should first be made a thorough examination of the mouth as a whole, with a view to detemining what is necessary to put it is first-class efficient condition. The examination should not be confined to the decay of the teeth alone, but to all the adjacent soft tissues and to the occlusion and contact points.

The examination having been made, the conditions should be explained thoroughly to the patient. This can best be done by the aid of models, fillings, and inlays on "show up work," etc., together with the pointing out of the differences between good and indifferent work.

In this connection, the wise dentist will find it of benefit to keep thoroughly posted on his physiology and to familiarize himself with short, brief, pertinent statements which will impress upon the patient the importance of a perfect masticatory apparatus.

The masses of the people do not as yet realize the importance to good health of sound, clean, efficient teeth. Most people have the erroneous idea that attention to their teeth is a very insignificant matter compared to other things to which they give attention for the promotion of their general health. For his own personal advancement and reward, and for the advancement of the prestige of his profession, the dentist should not lose an opportunity to acquaint his patients with the facts concerning the effects on general health of the condition of the teeth and mouth.—*American Dentist.*

*Cause of Breakage of Platinum Pins In Teeth.*

The breaking of platinum pins is invariably caused by bringing the pins into contact with some base metal or substance such as iron, zinc, lead, or carbon, which converts the tough and fibrous metal into a crystalline and fragile material. The platinum which is used for the pins of teeth must be absolutely good or it could not be successfully drawn into wire and made into pins, hence when a pin breaks and shows a granular or crystalline fracture it is due to want of care in manipulation. Traces of wax left upon the teeth, especially cheap wax which contains impurities or gum, will when heated quickly carbonize, and as platinum readily absorbs carbon, a crystalline fracture is easily brought about. The same remark applies to cheap solders, which should be avoided; they contain base metals which make them very fusible but at the same time most injurious to the platinum pins of teeth.—*Claudius Ash & Co., Limited.*

*Dental Reflex Pain in Women.* Hyperæmia and anæmia are frequently associated with facial and dental neuralgia. It is a well known fact that some nervous affections are ushered in with odontalgia. Even pelvic conditions will produce odontalgia reflexly.

Ladies often complain of toothache and painful gums during or shortly before or after the menstrual period, especially about the teeth being sensitive to heat or cold.

If carious teeth are present, menstruation can not be looked upon as the cause of the aching, but a great many women with carefully kept mouths and sound teeth will complain of toothache. In such cases it is almost a positive proof that the pain is caused from high blood pressure.

It is true we find more extensive caries during pregnancy than at other times. Why? Not because the teeth are worse during gestation than they were before conception, but because patients go into pregnancy with badly kept mouths. Caries no doubt existed, but the dentist was not consulted because the patient had no toothache. A woman, especially if she be married, ought to be as careful about her teeth as she is about her general health. Good health always means a clean mouth.—Dr. C. J. Spain in *Western Journal.*

*Tropacocain in Spinal Anesthesia.* Dr L. L. Stanley reports in the Journal of the American Medical Association, April 8, 1916, the results of 280 cases of spinal anesthesia with entirely successful results. While the technique is somewhat complicated it has been used with marked success for very serious operations. In fact the author reports only two failures in 400 cases, and these were most unfavorable cases for any anesthetic. In the 280 cases reported the following conclusions are reached

No fatalities, only slight shock, only 8 per cent. of headaches, no pneumonia, few postoperative complications, no paralysis, brief convalescence, muscular relaxation, no alarming fall of the blood pressure, no impairment of pulse rate, sufficiently prolonged anesthesia for the average operation.

*Flasking.* Before pouring the plaster of Paris into the flask to form the mould, thoroughly dust the inside of the flask with French chalk to prevent the plaster sticking to it. The vulcanized case can then be quite easily removed without bruising the flask.—*Claudius Ash, Sons & Co., Limited.*

*Medical College Attendance.* For the session of 1914-1915 there were enrolled in 82 medical schools in this country 12,976 students. The average income per student was $150, whereas the average expense was $419. The students paid approximately two million dollars, while the expense of conducting the schools was nearly five and one-half million dollars. In one school about $140,000 were paid for salaries and in another only $3,600 were paid out for teachers' salaries.

*Crown and Bridge Work and Partial Plates.* The importance of constructing artificial dentures upon a logical basis and with a correct conception of the principles underlying the selection of artificial teeth, is more apparent now than at any time in the immediate past. We are just beginning to appreciate the truisms that the covering of tooth crown with a gold shell to act as a bridge abutment or the devitalization of a tooth, marks tragic epochs in man's life. Just in proportion as some forms of crown and bridge work were lauded in years past, and still are at the present time in some quarters, as the ideal method of dental substitution, just in that proportion must they be condemned today as a prolific source of systemic and local disturbances of degrees of severity impossible to appreciate, unless we are willing to avail ourselves of the diagnostic possibilities of bacteriology and radiology. Crown and bridge work is being made to relinquish the high place it has most unwarrantedly occupied in the field of prosthesis in favor of the full or partial denture the least dangerous to the general health of the patient and the most satisfactory form of dental prosthesis if constructed upon the scientific principles which have been given to the profession by men whose names should be household words wherever dentistry is practiced.—Julio Endelman in *Pacific Gazette.*

*Chronic Poisoning by Emetin.* In a preliminary note, H. H. Dale (*Brit. Med. Jour.,* Dec. 18, 1915,) cites some clinical observations of instances in which the long continued use of emetin in doses which were individually harmless apparently led to the development of toxic symptoms. The symptoms observed included diarrhea and general toxic manifestations, and suggested the possibility of cumulation. Experiments made on cats and rabbits showed that the repeated administration of subtoxic doses invariably led to cumulation with the development of diarrhea, lethargy, somnolence and even coma. Post mortem examination of these animals showed intestinal irritation and damage to the kidneys and liver. These animal observations together with the clinical experiences led to the warning not to indulge in the indiscriminate use of emetin beyond the limits already established as the result of expert observation.—*Journal Allied Societies.*

*N. Y. Dental Clinics.* All the free clinics, including those for treatment of diseases of the nose and throat, that were under the auspices of the City of New York, were abolished on January 1st, 1916, by the Department of Health. One exception only was made, the dental clinics being retained, and these have been moved into school buildings and can no longer be classed as community clinics.

This latter move by the Board of Health should bring good results as regards better care for the mouths of the public school children. Prophylactic work can be more thoroughly done, there will be no difficulty in following up cases and children will lose no time in transportation, under the new conditions. The same equipment will be in use as formerly.—*Journal Allied Societies.*

*Health Diet.* There are a few foundation principles which, if thoroughly understood, any of you can work out a proper diet for himself.

1st. Rest for the digestive organs.

2d. The quantity of food required.

3d. The various classes of food, their purpose and value.

4th. The combinations of foods.

1. Rest is nature restorative. We require seven to nine hours bodily rest in the twenty-four, and if we overwork for a few days, we find it necessary to take a whole day's rest in order to get back to normal.

When the digestive organs have been overworked for a time, it is just as necessary that they have a rest. We should not eat unless there is a real appetite. If you feel bad in any way, do not eat. In place of the meal, take the juice of half a lemon in a glass of hot water, nothing else, but drink all the water you want, either cold or hot, between that and the next meal time.

The omitting of one or two meals should not be called a fast. After you have gone without food for twenty-four hours you ought to stop work. If you are to fast longer than one day, have the advice of a physician.

2d. The quantity of food that each person requires is a hard question to answer. Most persons who have reached adult life have overeaten so much of the time and the stomach has become so distended that if they eat to a sense of full satisfaction they will have taken a third to a half more food than the body requires. Therefore the appetite, like the conscience, needs to be carefully educated to right values. If you feel logy and lazy, cut down on the food supply. If there is a bad taste in the mouth on arising in the morning, reduce the supply still more. Certainly every dentist cleans his mouth before retiring, so if the breath is bad in the morning, it must come from the stomach. With a little careful watching you can soon stop over eating if you appreciate the feeling of physical well being more than the satisfaction of a full stomach.

3d. Foods are divided into two classes. Proteids or albuminous substances, and the carbohydrates and fats. The proteids are represented by the meat products and in the physiological economy replace the waste tissue. The carbohydrates, which are the starches and sugars, and the fats, furnish the body with heat and working energy.

When more proteid food is taken than is needed to replace the tissue waste, it is a real burden on the organs of elimination, for this waste must be thrown off.

Fats and carbohydrates when oxidized in the body are ultimately burned to simple gaseous products, viz., carbonic acid and water. Hence these waste products are easily and quickly eliminated. On the other hand, with the proteid foods, the story is quite different. These substances, when oxidized, yield a row of crystalline nitrogenous products which ultimately pass out of the body through the kidneys, frequently burdening these organs beyond endurance.

4th. The combination in which foods are taken into the stomach is of the utmost importance. It has been proven that meat digests best when the stomach secretions are strongly acid and also that the stomach provides this excess of hydrochloric acid when meat is injected.

Again, the digestion of starch begins in the mouth when it is thoroughly mixed with the alkaline secretion of the salivary glands. When this mixture is taken to the stomach, it partially neutralizes the acid content of the gastric juice. Hence if meat is eaten at the same meal, it has less opportunity for proper digestion.

With these facts before us, let us make some practical deductions from them.

Meat once a day will furnish the body with sufficient proteid, and this should be eaten in combination with cooked non-starchy vegetables and a vegetable salad. The starches may be eaten with any of the cooked vegetables or with the sweet fruits, and with bacon or eggs, but not with the acid fruits, such as grape fruit, oranges, apples, etc.

Milk and cream may be used with all the fruits.—Dr. S. E. Johnston in *Western Journal.*

*Teething as a Cause of Symptoms in Infants.* The problem of dentition in infants in one shape or another reappears from time to time in medical literature all the world over, and mothers and nurses appear to be, as a correspondent of the *Lancet* points out this week, in entire agreement that teething is the *fons et origo* of that miscellany of ailments to which infants during the period of dentition are peculiarly prone. It may further be asserted with some confidence that the majority of family practitioners are of the same opinion in a somewhat restricted sense. The dissentients from this view are mainly to be found in the ranks of hospital physicians and specialists, but even among pædiatric physicians of the first rank there are diversities of opinion on this thorny question. To give a few instances only, Dr. A. Jacobi, of New York, a pronounced sceptic and the Nestor of Pædiatrics, caustically remarks in his "Therapeutics of Infancy and Childhood" (p. 315): "There are fortunately, practitioners who prefer making a diagnosis of the real condition of the ailing baby, and that and its improvement or cure comprise the main treatment I recommend for 'difficult dentition.' " Dr. Leonard Guthrie, whose opinion both as a neurologist and as a specialist in the diseases of children deserves the most respectful hearing, devotes in his "Functional Nervous Disorders in Childhood" an entire chapter to the disturbances associated with primary dentition. He discharges whole broadsides of good-natured chaff at the expense of those who believe that the physiological process of dentition has pathological consequences. He says on p. 173: "I believe, therefore, that the ordinary phenomena of painful dentition are dependent on alimentary disturbances associated with a 'common cold,' and that dentition is painful because the gums become unhealthy." Dr. G. F. Still, in a wholly admirable article on the same subject in his "Common Disorders and Diseases of Childhood," gives a long list of the symptoms which he ascribes to the process of teething, and concludes with the words: "I am inclined rather to enlarge than to restrict my own conceptions of the role of dentition in producing disturbances of various kinds in infancy." Sir James Goodhart, in his "Diseases of Children" (seventh edition), says: "Increased activity of all the physiological processes at work necessarily implies greater risks of friction between one organ and another, or even of regular breakdown. Excessive energy, if not properly regulated or adequately expended, is liable to lead to an explosion of some sort or another. With such divergences of opinion between experts of unquestioned authority, how shall lesser men decide? It may, however, be remarked that the majority of authorities who write, at any rate authoritatively, on this problem and who as teachers have great influence in formulating opinion among practitioners, acquire the larger proportion of their knowledge from so-called pathological experiences, for the greater number of children and infants whom they see are either sick or ailing. On the other hand, it is interesting to note that the opinion of that now considerable number of medical men and women who act as officers to Infant Welfare Centres is almost unanimously in favor of the belief that teething is a common cause of disturbance to health; this fact is instructive, because at "Infant Consultations" the vast majority of the infants are relatively healthy. It has been claimed that dentition is a physiological process, and that since no physiological process should be accompanied by pain, dentition itself can not be the cause of pathological phenomena. This syllogism is unsound in its major premise, in its minor premise, and in its general conclusion. There is no true distinction between a physiological and a pathological process; both are designed to preserve life in the manner best suited to meet the existing conditions of the environment. If they should happen to be accompanied by pain or other disturbance of bodily harmony so much the worse for the individual. Pain is one of the most efficient means of protecting the body against existing dangers, and is in essence physiological. Parturition is a physiological process and a distinctly painful one, especially in the case of the contracted pelvis; and so also may be the process of dentition in the contracted jaw of civilization, as many can avow from personal memories of the cutting of the wisdom teeth. During the period of dentition many symptoms develop either as a consequence of, or coincident with, the eruption of each tooth which must give the careful observer much food for reflection. The almost invariable flush on the cheek of the healthy child, the running of the nose or the lachrymation in those less fit, the pharyngeal cough, the bronchitis or the diarrhea in those of still poorer condition, are best capable of explanation as the misdirected incidence of nervous energies, whether reflexly or by irradiation engendered by the eruption of the tooth, taking effect in organs or in situations of least resistance. This may possibly explain why when any particular function has previously been disturbed there is apt to be a recurrence of the event during the period of dentition—why, in other words, the child always cuts its teeth "with bronchitis or diarrhea," as mothers and nurses are wont to believe.—*London Lancet.*

*Dental Libraries.* The following editorial taken from the *International Journal of Orthodontia,* is in large measure true as applied to dentists; but we happen to know of at least eight dental schools with excellent libraries that are in constant use.

"Tell me what you read, and I will tell you what you are." That may apply to the dentists and, if we should judge them by their libraries, we would find that very few of them would be classed as dentists. In some dental offices we find quite a collection of books on subjects not relating to dentistry, but, taken as a whole, the dental and medical books are very few. Occasionally there are dentists who possess dental libraries, and these dentists are divided into two classes—the man who buys dental books to read and the man who buys books only to place on shelves. We know of several large dental libraries owned by private individuals who have made it a habit to collect dental books, but who fail to make it a habit to read them. Of course, these individuals are to be commended for their part, as in a few years the libraries will probably pass into the hands of some institutions which will make use of them.

The dental library in the dental college is practically a thing that exists only in name. It is true that dental colleges receive volumes on dental subjects from publishers, as specimen copies of such books are sent to the colleges gratuitously, to be reviewed and placed in the libraries. The college library is, however, generally given a place in the private office of some individual—the dean or secretary of the school—and carefully guarded from the eyes and hands of the student. Consequently, so far as the student body is concerned, the library in a dental college is practically of no value. There are few schools that make an attempt to procure a library for the benefit of the students, and the selection of books is, as a rule, very poorly planned. In fact, the greater number of the books usually found in these libraries will be on the subject in which the dean or secretary is particularly interested. If either of these individuals is interested in one or two special subjects, the remainder of the collection of books is very poor. Works on anatomy, physiology, and chemistry are decidedly rare in a dental library, while they should be the basic subjects on the student's course. When we consider books on oral surgery and orthodontia, or subjects which may be termed specialties of dentistry, we find those subjects are more neglected than any other.

While we realize that the question of a dental library in the dental college is a rather new phase of education, it is nevertheless a feature of educational work that must be considered, and the future of dental education depends to a certain extent on a properly selected reference library that is accessible to the student.—Editorial in *International Journal of Orthodontia.*

*Why Not Live Longer?* Dr. F. J. Will, the Medical Director of the Bankers' Life Insurance Company, is the writer of an article entitled: "Why Not Live Longer?" "If you love life and would lengthen your days of living, the best thing you can do is to 'slow up' on your eating and drinking. High living and overeating shortens many lives. Many people are eating themselves to death. Eating too much and not exercising enough might be called slow living, but it is just as deadly as fast and high living. It is not necessary to burn the candle at both ends in order to become a shining mark for the grave. High living and lack of exercise demand as many victims as any foes of mankind. A person may lead an exemplary life, but if he eats too well and does not take the precaution to exercise, he is sooner or later called upon to pay the penalty. Food is fuel. If you continue to choke the furnace with more fuel than it can consume, trouble is sure to follow.

"The dangers of overeating are emphasized in reviewing the ten leading causes of death. While a large share of the nervous disorders responsible for the untimely deaths are due to living on a six-cylinder basis, in the effort of trying to do a four-horse power business with a two-horse power body, back of these, wasted nerves and energies in a great many cases, is an overloaded stomach.

"High among the leading causes of death are diseases of the nervous system and at the head of these are apoplexy and cerebral hemorrhage, which practically are the same. These are due to high living more than to any other cause. They are aggravated by fast living, liquor and late hours, but the principal cause is overeating. The high liver, the man who likes midnight suppers, wines and big dinners, the man who likes to gorge on big, thick, 'juicy' steaks with highly flavored side dishes prepared by a chef, who can make hemlock bark palatable, is a marked man unless he exercises.

"Excessive meat eating, without exercising, is one of the most serious causes of apoplexy."—*Western Dental Journal.*

*Aseptic Canal Treatment.* Shall the average dentist earn the title of a humanitarian and philanthropist by doing aseptic canal work, or shall he employ such scientific technic as his and his patients' means permit? What are patients in moderate circumstances to do who can not have molars treated and filled by "aseptics?" For such operative work the dentist usually requires three or four hours, and charges five to twenty-five dollars per hour. As a consequence, many patients must go to the men who fill canals with pastes. Is it not possible that those who use such pastes and highly advocate them have good reasons for doing so? Do they not think that such pastes are the best they can afford to give the patient for the compensation they are to receive? Do they not believe these preparations shield their septic methods and careless ways of treating root canals? Can they be blamed very much for so doing? Circumstances certainly alter cases. Let those who use canal pastes truthfully analyze these facts. Let the average dentist do likewise.—Samuel Lang in *Items of Interest.*

